# Mast Cells in Gut and Brain and Their Potential Role as an Emerging Therapeutic Target for Neural Diseases

**DOI:** 10.3389/fncel.2019.00345

**Published:** 2019-07-30

**Authors:** Giovanna Traina

**Affiliations:** Department of Pharmaceutical Sciences, University of Perugia, Perugia, Italy

**Keywords:** mast cell, neuroinflammation, pain, stress, depression, gut–brain axis, probiotics

## Abstract

The mast cells (MCs) are the *leader* cells of inflammation. They are well known for their involvement on allergic reactions through degranulation and release of vasoactive, inflammatory, and nociceptive mediators. Upon encountering potential danger signal, MCs are true sensors of the environment, the first to respond in rapid and selective manner. The MC activates the algic response and modulates the evolution of nociceptive pain, typical of acute inflammation, to neuropathic pain, typical not only of chronic inflammation but also of the dysregulation of the pain system. Yet, MC may contribute to modulate intensity of the associated depressive and anxiogenic component on the neuronal and microglial biological front. Chronic inflammation is a common mediator of these co-morbidities. In parallel to the removal of the etiological factors of tissue damage, the modulation of MC hyperactivity and the reduction of the release of inflammatory factors may constitute a new frontier of pharmacological intervention aimed at preventing the chronicity of inflammation, the evolution of pain, and also the worsening of the depression and anxiogenic state associated with it. So, identifying specific molecules able to modify MC activity may be an important therapeutic tool. Various preclinical evidences suggest that the intestinal microbiota contributes substantially to mood and behavioral disorders. In humans, conditions of the microbiota have been linked to stress, anxiety, depression, and pain. MC is likely the crucial neuroimmune connecting between these components. In this review, the involvement of MCs in pain, stress, and depression is reviewed. We focus on the MC as target that may be mediating stress and mood disorders via microbiota–gut–brain axis.

## Introduction

A *basal* inflammation is a protective condition. In physiological levels, both pro- and anti-inflammatory mediators may result essential for induction and maintenance in neuroplasticity phenomena (Bezzi et al., 1998; Avital et al., 2003), whereas in high doses they can cause an acute inflammation that, in turn, can produce a state of chronic inflammation and achieving neural diseases (Dong et al., 2017; Kempuraj et al., 2017). An inflammation state is underlying pain, stress, and depression (Hendriksen et al., 2017; Gupta and Harvima, 2018).

The International Association for the Study of Pain (IASP) defined the pain as “*an unpleasant sensory and emotional experience associated with actual or potential tissue damage, or described in terms of such damage*” (Iasp, 1979). According to this definition, it emerges that pain, originating from peripheral damage, is progressively enriched by neuropsychological and emotional components, which born in the brain and modulate their perceptive and purely subjective components (Mannion and Woolf, 2000). A chronic pain is frequently the first determinant of psychological and mood disorders (Humo et al., 2019).

Stress is a complex dynamic condition in which homeostasis is altered or threatened (Rea et al., 2016). Stress and inflammation represent the main pathogenic factors in multiple diseases that are often comorbid including fibromyalgia syndrome, migraine, as well as irritable bowel syndrome (IBS) (Bennett et al., 1998; Tak et al., 2011). Stress, particularly in early stages of life, is one of the main predictors of the onset of major depression disorder and chronic pain, and it may affect the perception of the pain and exacerbate it (Liu and Chen, 2014; Burke et al., 2017; Humo et al., 2019). Studies point out that major depression is prevalent in patients affected by chronic infections and suggest that a chronic inflammation can increase its incidence (Hauser et al., 2011). Elevations in pro-inflammatory cytokines have been reported in patients suffering from depression and chronic pain (Martinez et al., 2012).

Major depression, emotional, and chronic stress lead to the activation and alteration in limbic regulation of the hypothalamic–pituitary–adrenal (HPA) axis, whose altered regulation is usually associated with centralized pain syndromes (Menke, 2019). Under normal conditions, an acute stress induces the hypothalamus to release the corticotrophin-releasing factor (CRF) that induces the anterior pituitary gland to release adrenocorticotropic hormone (ACTH), which causes the adrenal cortex to release glucocorticoids that play metabolic roles (Herman et al., 2003). A negative feedback loop turn off the HPA axis activation (Kageyama and Suda, 2009). Subjects with pain syndromes present altered signaling from HPA axis but also mood disorders, including depression and anxiety (Bao and Swaab, 2019). The crucial link connecting these disorders is the inflammation mediated and modulated by cells, whose *leaders* are the mast cells (MCs). MCs are versatile cells that serve important functions in both innate and adaptive immunity surveillance, and the first responders to insults. They are equipped with extraordinary functional peculiarities and respond strongly to HPA axis activation (Heron and Dubayle, 2013; Skaper, 2016; Theoharides, 2017; Gupta and Harvima, 2018).

Increasing evidence has pointed to the relationship between intestinal microbiota and brain, showing that the gut inflammatory *milieu* may play a crucial role in the induction of several nervous conditions including stress, anxiety, and depression as well as in neuroinflammation (Sherwin et al., 2016; Cussotto et al., 2018).

From various studies it emerges that the MC is particularly responsive to microbiota conditions and its stabilization through appropriate combinations of probiotics could represent a new potential therapeutic tool to control neural disorders that underlie its activation (Wouters et al., 2016). Therefore, the goal is to stabilize the MC, and do it starting from the intestine.

In this review, we focus on the MC as potential target that may mediate neural diseases via microbiota–gut–brain axis.

## Overview on Mast Cells

Mast cells are heterogeneous and ubiquitous cells of the vascularized tissues where they work as immune gatekeepers at host/environment interfaces, like the skin, airways, gastrointestinal, and urogenital tracts to respond to different allergens, pathogens, parasites, and other danger agents that can be ingested, inhaled, or encountered after breakdown of the epithelial barrier. In addition, MCs organize the inflammatory response, modulating the quality of tissue repair and remodeling at the same time (Traina, 2017; Forsythe, 2019).

Mast cells originate from hematopoietic-derived immune CD34+ multipotential stem cells in the bone marrow and circulate in the blood in low numbers as immature precursors. They migrate to locate in mucosal and connective tissues completing their differentiation in mature MCs on the influence of local residing microenvironment, which defines their phenotype and, consequently, their function (Beaven, 2009; De Zuani et al., 2018). In particular, MCs represent about 2–3% of the immune cellular pool of the lamina propria, and in the muscular and serous layers (3,000–25,000 MCs/mm^3^), located in strategic position in proximity of blood, lymphatic vessels, and nerves (Irmak et al., 2019). Stem cell factor (SCF) binds to c-kit tyrosine kinase receptor of MCs and it is a necessary component for their survival, proliferation, and differentiation (Okayama and Kawakami, 2006).

In small quantity, MCs are also present in the brain. Here, they are located in area postrema, parenchyma of thalamus and hypothalamus, leptomeninges, pineal organ, infundibulum, choroid plexus, and in *dura mater* of the spinal cord. Their interaction with meningeal afferents and dural vasculature may have a crucial role in migraine headache (Rozniecki et al., 1999; Xu and Chen, 2015; Arac et al., 2019). In the brain MCs are located on the abluminal side of blood vessels, where they interact with neurons, glia, and endothelial cells (Hendriksen et al., 2017). The total number of MCs present in the central nervous system (CNS) is limited and it is difficult to calculate it because subject to changes related to age, sex, and animal species and also in response to outside environmental conditions (Silver and Curley, 2013). In human healthy brain, in meninges and perivascular area <5 MCs in 5 μm thick tissue sections were found during autopsy (Maslinska et al., 2005). During infection MC numbers increase to 11–20 in meninges and 5–10 in perivascular area. In mice brain, MC numbers are increasing from 150 to 500/50 μm sections thick during development (Nautiyal et al., 2012). But they are very powerful cells and even few MCs are able to release a sufficient amount of inflammatory mediators that can affect the blood–brain barrier (BBB) integrity and, in turn, activate glia and neurons (Hendriksen et al., 2017). All the unique features of MCs allow them to start, amplify, and prolong the inflammatory response. MCs are armed with a vast repertoire of receptors to ligands. MCs possess high affinity receptors, Fc𝜀RI for immunoglobulin E (IgE) binding protein on the cell surface and cytoplasmic proteases-containing granules under the influence of molecules, such as SCF, interleukin (IL)-3, and IL-9, as well as bacterial-derived molecules [lipopolysaccharide (LPS) and peptidoglycan] (Bischoff and Kramer, 2007; Frossi et al., 2017). In this manner, MCs can be activated by pathogens, antigen-bounded Ig, and also by soluble or physic factors, drugs, temperature, pressure via transient-receptor-potential channel 2 (TRPV2). Their receptors include also complements receptors, Toll-like (TLRs, 1–7, 9), nucleotide-binding and oligomerization domain (NOD)-like as well as receptors for cytokines and microbe associated molecular pattern receptors (MAMPs) (Zhang et al., 2012; Traina, 2017).

Interestingly, MCs have the receptors for CRF, strengthening link between stress and MC (Traina, 2017; Kempuraj et al., 2019). In particular, MCs possess both CRF1 and CRF2 receptors, indicating their central role in visceral hyper-sensibility, in sensitizing sensory nerve terminations and, possibly, in lowering pain thresholds (Eller-Smith et al., 2018). In addition, they contain vast CRF stores (Theoharides and Konstantinidou, 2007). A recent study showed that CRF2 represents a negative modulator of MC activation, suggesting a crucial homeostatic role of MC in the CRF system and in the disorders associated with his (D’Costa et al., 2019).

**FIGURE 1:**
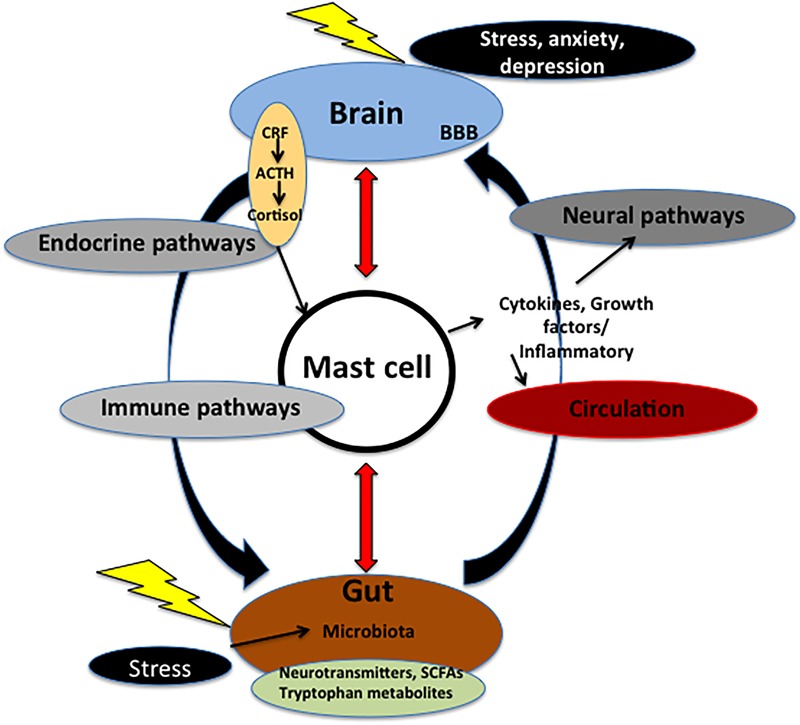
Microbiota–gut–brain axis. The axis is fundamental for the regulation between the brain and the gut. Communication within this axis involves the coordination of different factors and systems. It includes CNS, circulation, and endocrine and immune pathways. Mast cell (MC), emblem of the neuroimmune network, acts at the crossroad of intestinal mucosa, microbiota, and nervous system. Microbiota may control events both in peripheral and CNS via nerve activation, cytokine production, neurotransmitter and SCFAs release, and through systemic circulation. These signals may pass through BBB inside the brain, and, in turn, activate MCs and microglia involved in immune surveillance. In addition, in the brain these molecules control different functions, such as the regulation of HPA axis. This axis regulates various activities, including MC activation. Such activation causes the release of cytokines and growth factors that, in turn, can act both centrally and peripherally. Chronic stress and depression lead to alteration of the HPA axis as well as dysfunctions in the microbial gut environment and pain visceral signaling. On the other hand, in the gut mucosal MCs are influenced by microbiota *milieu* and by visceral conditions. ACTH, adrenocorticotropic hormone; BBB, brain–blood barrier; CRF, corticotrophin-releasing factor; SCFAs, short chain fatty acids.

Mast cells own a great number of co-stimulatory molecules. These mediators include members of the tumor necrosis factor TNF/TNFR families, which allow them to interact with different cells populations and with bacteria and fungi through the expression of different pattern-recognition receptors (PRRs) (Frossi et al., 2017; De Zuani et al., 2018). MCs harbor a large amount of granules that contain preformed and *de novo* synthesized molecules. Preformed mediators include histamine, serotonin (5-HT), proteases, heparin, and growth factors including TNF-α, proteoglycans, initiating the early recruitment of immune cells at the infection site. Newly synthesized mediators consist of lipid-derived mediators, such as prostaglandins and leukotrienes and cytokines (IL-1β, IL-2, IL-4, IL-6, IL-8, IL-16, and IL-18) that affect the physiology of neighboring cells (Hendriksen et al., 2017; Traina, 2017). Activation of MCs via one of the pathways can release a plethora of pro-nociceptive mediators. MCs are able to respond to activation of the principal stress system, HPA axis and, in turn, pro-inflammatory cytokines are potent stimulators of the HPA axis (Theoharides, 2017). Their aberrant activity may also give to neurodegenerative and mood disorders (Hendriksen et al., 2017). In addition, mediators released by MCs may affect epithelial integrity and viability (Albert-Bayo et al., 2019). MCs represent the connecting link between brain and immune system, because they respond and release neurotransmitters and immune molecules (Forsythe, 2019; Figure 1). Finally, in the brain MCs does not possess IgE receptors (Fc𝜀RI) (Theoharides and Konstantinidou, 2007). So, the brain does not manifest allergic reactions (Forsythe, 2019).

## Mast Cells and Pain

Both peripheral and CNS participate in pain. CNS is involved not only in spino–thalamic–cortical pain system, but also in the limbic areas, which modulate the component emotional-affective of pain, as well as in the cognitive areas, which modulate adaptive, motivational, and relational aspects (Mannion and Woolf, 2000). Headache is one of the main symptoms associated with these conditions (Irmak et al., 2019). Evidence shows the involvement of MCs on pain and stress (Eller-Smith et al., 2018). In stress condition, activation of HPA axis induces an increase of CRF release that could result in MC activation and in sensitization of nerve terminals increasing pain signaling (Eller-Smith et al., 2018). MCs and nerves communicate bi-directionally. Calcitonin gene-related peptide (CGRP) released from meningeal nociceptors may degranulate MCs that release histamine that, in turn, activates mechanosensitive C-fibers that release CGRP and substance P (SP) (Julius and Basbaum, 2001; Irmak et al., 2019). Actually, CGRP co-localizes with SP and both neurotransmitters were found adjacent to mucosal MCs. MCs sustain peripheral neurogenic inflammation through the further release of SP and CGRP, that perpetuate inflammatory molecule release (Julius and Basbaum, 2001). Afferent fibers express the receptors involved in nociception, such as transient receptor potential vanilloid 1 (TRPV1), transient receptor potential ankyrin 1 (TRPA1), and proteinase-activated receptor 2 (PAR2) (Kim et al., 2010). The activation of PAR2 starts downstream sensitization of TRPV1 and TRPA1 involved for the generation of visceral hyper-sensibility (Amadesi et al., 2006; Irmak et al., 2019). PAR-2 is expressed by dorsal root ganglia that co-express TRPV1, TRPV4, TRPA1, and SP and CGRP, is activated by tryptase and SP, and its activation can result in neurogenic inflammation (Amadesi et al., 2006). TRPV1 is an intriguing target for pain control in MC-dependent disorders (Eller-Smith et al., 2018). A possible role of histamine in TRPV1 activation has been suggested (Kajihara et al., 2010; Eller-Smith et al., 2018). SP induces CRF receptor expression on MCs (Asadi et al., 2012). MCs also synthesize nerve growth factor (NGF) that in autocrine mode stimulates the MCs to release pro-nociceptive mediators (Eller-Smith et al., 2018). NGF binds to its receptor TrkA evoking pain hypersensitivity (Eskander et al., 2015).

Evidences suggest that MCs may upgrade a cascade of inflammatory events that result in trigeminal activation (Levy et al., 2006; Irmak et al., 2019). MC releases nitric oxide (NO), crucial mediator of persistent neuronal damage triggering neurogenic inflammation (Ramachandran et al., 2014; Ramachandran, 2018). CGRP and mediators released from MCs induce meningeal vasodilatation and activation of sensory nerve fibers (Irmak et al., 2019). In addition, the nucleotide adenosine triphosphate (ATP) directly excites trigeminal nerve terminals and degranulates MCs, suggesting that ATP may contribute both to excitation and to meningeal neuroinflammation in the unit of dural MCs and trigeminal afferent fibers (Koroleva et al., 2019). A fast inhibitory interaction between respective receptors could revert MC-derived neuroinflammation in sensory nerve endings and influence their time course activation.

## Mast Cells and Stress

The exposure to chronic stress may induce irreversible modifications in the brain regions responsible for perception of pain (Fasick et al., 2015).

Specific areas, including hypothalamus, amygdala, prefrontal cortex, and hippocampus, and their interaction with limbic system are involved in response to stress (Lupien et al., 2009). A crosstalk between MCs and microglia in these areas could explain stress-induced inflammation (Hendriksen et al., 2017; Traina, 2017). Stressful conditions can also activate peripheral MCs, and increase glial activation (Dong et al., 2017). The integration of these centers results in the activation of HPA and in autonomic nervous system (ANS) that modulates also enteric nervous system (ENS), inducing an exacerbation of inflammatory conditions (Rea et al., 2016). Prolonged neuroinflammation has deleterious effects that involve changes in brain parenchyma, BBB alterations, neuronal hyper-excitability, and neuronal death (Hendriksen et al., 2017). The microglia and MCs become hyper-activated, realizing IL-1β, IL-6, and TNF-α (Skaper, 2016; Kempuraj et al., 2017; Traina, 2017). Variety of adhesion molecules, cytokines, chemokines, and metalloproteases contribute in the development of inflammatory response in brain through the degradation of extracellular matrix and tissue remodeling (Hendriksen et al., 2017). Acute stress condition increases BBB permeability resulting in ion unbalance, entry of immune molecules, and instable CNS environment (Hendriksen et al., 2017). The penetration of reactive T cells into the CNS is under the influence of MCs (Silverman et al., 2000). In stress conditions, increased peripheral CRF release consequent to dysregulation of HPA axis activation results in sensitization of nerve terminals (Eller-Smith et al., 2018). A prolonged increase of glucocorticoid levels is associated with a reduction of hippocampal volume and impairments in memory, perception, and attention (Bremner, 2006). Hippocampal volume reduction may be due to high levels of glucocorticoids that damage mature neurons or rather to high levels of cortisol that suppress neurogenesis (Bremner, 2006). Also a recurrent depression causes a volume reduction of the hippocampus (Bremner et al., 2008).

## Mast Cells and Depression

Studies indicate that major depression is prevalent in patients affected by chronic infections suggesting that a chronic inflammation condition can increase depression incidence (Hauser et al., 2011). Major depression disorder attend changes in monoaminergic neurotransmission, imbalance of excitatory/inhibitory signaling, hyperactivity of the HPA axis, abnormalities in neurogenesis (Milenkovic et al., 2019). The disorder was accompanied to increase of circulating IL-1β, IL-6, and TNF-α (Marini et al., 2016). Administration of inflammatory cytokines leads a major depressive disorder condition like to the one induced by stressor agents (Anisman and Hayley, 2012).

Proinflammatory cytokines can induce the activation of indoleamine 2,3-dioxygenase (IDO), enzyme involved in the catabolism of tryptophan to kynurenine and higher levels of kynurenine are linked with a depression condition (Gabbay et al., 2012). Some studies reported that the prevalence of major depression and other neurological and psychiatric symptoms such as anxiety, sleep disorders, and headaches are detected in patients suffering of mastocytosis, a disease characterized by MCs accumulation and activation (Moura et al., 2011; Hendriksen et al., 2017). Such subjects present lower levels of tryptophan and 5-HT, and high IDO1 activity and kynurenine acid. It is know that MCs may be activated by kynurenine metabolites (Hermine et al., 2008; Kawasaki et al., 2014), whereas MCs mediators can affect the IDO pathway bringing to an imbalance between kynurenine and 5-HT ratio. Finally, pro-inflammatory cytokines increase monoamine reuptake by reducing 5-HT levels (Theoharides et al., 2015; Hendriksen et al., 2017). A different pattern of HPA axis activity has been described for atypical depression (Juruena et al., 2018).

## Microbiota–Gut–Brain Axis

In the gut, the functional unit established by MC–nerve interaction is a crucial component in the interplay in paracrine signaling (Albert-Bayo et al., 2019). Enteric neurons and vagal and spinal afferents express receptors for molecules released by MCs. These molecules stimulate nerve terminals, thereby modulating the firing threshold. Similarly, neuropeptides and neurotransmitters released by neurons stimulate MC secretion of mediators, which further activate neuronal receptors, supporting the maintenance of this neuro-immune interplay (Forsythe et al., 2012; Albert-Bayo et al., 2019). The intestinal microbiota is composed of trillions of microorganisms, 10 times more numerous than the eukaryotic cells that make up the body (Rea et al., 2016).

Many studies support the relationship between complexity of the dynamic ecosystem of microorganisms that harbor the gut and health status (Eckburg et al., 2005; Cryan and Dinan, 2015). Under physiological and homeostatic conditions the microbiota helps to maintain a multitude of functions such as intestinal peristalsis, control of several metabolic functions, epithelial barrier integrity, pH balance, and immune priming and protection versus invading microorganisms (Kelly et al., 2017). Microbiota controls maturation and function of microglia (Nayak et al., 2014). The microbiota can influence emotional behavior through mechanisms that include microbe-derived bioactive molecules, immune and endocrine cell activation, and vagal nerve stimulation (Dinan and Cryan, 2013). The microbiota plays a crucial role in stress, anxiety, learning and memory, addiction, sexual behavior, social interaction, and depression as well as in neuroinflammation and neurodegeneration (Wang et al., 2016; Cussotto et al., 2018).

The *microbiota–gut–brain axis* is an integrate system that consists of a dynamic matrix of tissues and organs (brain, ANS, glands, gut, immune cells, and microbiota) communicating through a complex multidirectional manner via neural, endocrine, circulatory pathways in order to preserve homeostasis condition and resist to any perturbation to the system (Dinan and Cryan, 2013; Bermúdez-Humarán et al., 2019). Signals from the brain may influence the motor, sensory, and secretory functions of the gut and viceversa, visceral messages from the gut may influence brain function (Figure 1). Germ-free (GF) animals (without microbiota, born and maintained in sterile condition) present various immune disorders, including defective microglia (Erny et al., 2015).

Microbial metabolites and products of bacterial fermentation, such as short chain fatty acids (SCFAs), specifically acetate, propionate, and butyrate stimulate enteroendocrine cells to produce various neuropeptides, including neuropeptide Y and SP, that gain access to the circulation and/or receptor affecting ENS neurons or vagal innervation (Cani and Knauf, 2016; Figure 1). SCFAs together with other metabolites, such as polyamine, influence the immunity response and may restore physiological conditions in GF animals (Borre et al., 2014; Erny et al., 2015; Rook and Garrett, 2016).

Microbiota promotes tryptophan hydroxylase expression. In the gut, the amino acid tryptophan contributes to synthesis of bioactive molecules, including 5-HT (Agus et al., 2018). SCFAs and tryptophan transmit signals through interaction with enteroendocrine and enterochromaffin cells, and the mucosal immune system, cross the intestinal barrier, go in the systemic circulation, and may cross the BBB (Yano et al., 2015; Kelly et al., 2017; Martin et al., 2018). So, SCFAs and microbial regulation of tryptophan metabolism act as a link between microbiota and brain (Liu et al., 2018; Martin et al., 2018; Figure 1). Yet, microbiota produces a variety of other neuroactive molecules, including γ-aminobutyric acid (GABA), catecholamines, and acetylcholine, and may affect the HPA axis (Kelly et al., 2017; Wiley et al., 2017). 5-HT is a crucial substrate in the pathogenesis of mood disorders and intestinal microbiota may be a potential therapeutic target for 5-HT-related brain–gut axis diseases. Various species of bacteria are able of producing 5-HT, including *Streptococcus, Enterococcus, Lactococcus*, and *Lactobacillus* (Kelly et al., 2017).

Illness, stress, pain, or injury conditions can alter the microbial environment and induce to a large spectrum of effects including alteration of gut motility, loss of intestinal epithelial barrier integrity, antigen penetration, release of LPS into the bloodstream, mucosal MC activation, inflammatory mediator release, visceral hypersensitivity, and nociceptive sensitization, ranging from inflammatory bowel disease (IBD) and IBS to major depressive disorder (Bennett et al., 1998; He, 2004; Brzozowski et al., 2016; Rea et al., 2016). Subjects with IBD present an enhanced risk of anxiety and depression (Maunder, 2005; Goodhand et al., 2012). On the other hand, IBS is a complex disorder in which the inflammation is involved through gut–brain axis, resulting in altered neuroendocrine pathways. IBS is characterized by pain, visceral hyper-sensibility, intestinal microbiota imbalance, gut–brain axis dysfunction, and psychological disorders (Quigley and Craig, 2012). Microbial signals also modulate visceral pain anxiety- and depression-like behavior. In humans, significant changes in the microbiota have been noticed in a variety of amygdala-related clinical disorders, including depression and chronic visceral hypersensitivity. IBS patients present hyperactivity in the amygdala and closed brain regions in response to visceral stimulation (Barbara et al., 2007, 2011; Han et al., 2012; Cowan et al., 2018). Stress condition induces significant modifications in the composition of the microbiota. The mechanism of influence of stress on gut involves action of IL-6, IL-10, IL-1β, and TNF-α (Lew et al., 2018). It has been seen that norepinephrine enhances the virulence of some bacteria (Cogan et al., 2007). Tryptophan-regulating bacteria can function as antidepressant drugs (Dinan and Cryan, 2015). In conclusion, a dysregulation of the microbiota composition (*dysbiosis*) can start or exacerbate intestinal disorders as well as can influence emotional condition (Cryan and Dinan, 2015; Kelly et al., 2017).

## Mast Cells in the Gut

Mast cells are present in all layers within the gastrointestinal tract (Albert-Bayo et al., 2019). The close proximity of MCs and nerves is the emblem of the neuro-immune network and has indicated the existence of a bidirectional crosstalk between MCs and nerves acting in tandem with other neural and immune cells (Albert-Bayo et al., 2019). This dialog is crucial in the maintenance of intestinal homeostasis and it is responsible for diseases and in pain visceral perception (Quigley and Craig, 2012).

It has been observed that MC mediators are released in large quantity in gut of IBS subjects and leading to hypersensitivity of afferent neurons (Barbara et al., 2007). There is a growing literature to support the hypothesis that MCs perform a fundamental role in host–microbiota communication, by modulating the influence between them through changes in their activation (Wouters et al., 2016).

A brain–MC interaction is one conceivable mechanism linking stress responses and gastrointestinal symptoms with the involvement of vagal nerve pathway (Dong et al., 2017).

Chronic stress may lead to MC activation and modulate paracellular and transcellular permeability. In IBS an intestinal barrier dysfunction is implicated and the expression of tight junction (TJ) proteins is reduced in correlation with MC activation. Tryptase can activate PAR-2 on epithelial cells by increasing permeability through TJs (McDermott et al., 2003). Epithelial barrier breakdown is associated with an increase in pro-inflammatory cytokines, including IL-4, Il-13, IFN-γ, and TNF-α (Kim et al., 2018). Further molecules released by MCs, including chymase and prostaglandin D2, modulate epithelial chloride and water secretion and intestinal permeability (Wouters et al., 2016; Dong et al., 2017).

Microorganisms, such as bacteria and fungi, can induce MCs activation (De Zuani et al., 2018). Albeit some microorganisms can evoke a pro-inflammatory response, other microorganisms result able to reduce their activation, contributing to maintain their stability. This allows to limit or revert inflammation and to promote homeostatic conditions (Forsythe, 2016; Johnzon et al., 2016).

It has also been shown that MCs may phagocyte bacteria promising a scenario in which MCs may act as intermediate players between the microbiota and the adaptive immune system (Malaviya and Abraham, 2001).

In the gut, MCs are differentially functional in the different regions on the basis of local bacterial charge; in colon MCs have a greater abundance of TLR4 than the MCs present in the small intestine (Frossi et al., 2018). Bacterial challenge induces MC degranulation and release of mediators (Wesolowski and Paumet, 2011). An increase in histamine and tryptase secretion has been reported in biopsies from the gut of IBD patients (De Winter et al., 2012; Wilcz-Villega et al., 2013). These subjects show a lower bacterial diversity in the intestinal microbiota, an increase of the Proteobacteria phylum, and a decrease of Firmicutes (Halfvarson et al., 2017).

Mast cells interact with both the microbiota and the nervous system relating to enteric neurons through release of 5-HT, while are affected by SP or noradrenaline (Buhner and Schermann, 2012).

All these evidences support that MCs may substantially contribute to the balance in gut homeostasis, and their activation is linked to modifications and motor abnormalities and barrier dysfunctions (Barbara et al., 2007; De Zuani et al., 2018). In addition to well-established pharmacotherapy comprised of anti-inflammatories, antibiotics, and proton-pump inhibitors, valid treatment strategies on the microbiota may contain other options including probiotics, prebiotics, and food supplements with anti-inflammatory properties. The beneficial effects of probiotics have been recognized as therapeutic supplement in various disorders (Sarkar et al., 2018).

## Probiotic Challenge

Probiotics are defined as “*living microorganisms which, when administered in adequate amounts, confer a health benefit to the host*” (Hill et al., 2014). Probiotic supplementation is particularly useful for developing an understanding of the mechanism of action of selected bacterial strains, and a crucial factor in predicting the favorable health outcomes of nutritional intervention (Dinan and Cryan, 2013). Various studies reported that specific probiotic strains are able to counteract inflammatory conditions and exert considerable effects on immune cells and inflammation (Dominici et al., 2011; Bellavia et al., 2014; Persichetti et al., 2014; Tomasello et al., 2015a,b; Traina et al., 2016; De Marco et al., 2018; Sichetti et al., 2018). Gut microbiota composition may be effectively affected by dietary ingestion of probiotics and prebiotic, these last ones defined as non-digestible organic substances, capable of selectively stimulating the growth and/or activity of beneficial bacteria (Saulnier et al., 2013). Probiotics decrease plasma cytokine levels and reduce mitogen-stimulated cytokine in healthy subjects (Groeger et al., 2013).

Probiotics are recognized by TLRs in intestinal epithelial cells and immune cells. Probiotics can influence CNS function and modulate the HPA axis attenuating it, and altering the levels of corticosteroid and/or ACTH (Ait-Belgnaoui et al., 2018). HPA axis response to acute stress was attenuated by *Lactobacillus farciminis* (Ait-Belgnaoui et al., 2018). *Lactobacillus rhamnosus* and *Lactobacillus helveticus* influence brain-derived neurotrophic factor (BDNF) levels in basolateral nucleus of amygdala exerting an anxiolytic effect (Peng et al., 2019). Hippocampal *c-Fos* expression is modulated by *L. rhamnosus* and *L. helveticus* (Smith et al., 2014). *L. rhamnosus* reduces hippocampal expression of GABA receptor gene, suggesting a modulation of the balance of inhibition/excitation to control responses to stress, anxiety, and depression (Wiley et al., 2017). Lactobacilli and bifidobacteria are able to metabolize glutamate and produce GABA (Foster and McVey Neufeld, 2013; Wiley et al., 2017). Probiotics can modulate 5-HT, and dopamine, thus affecting both mood and behavior (Cowan et al., 2018; Hoban et al., 2018; Baj et al., 2019). The vagus and enteric nerves are significantly affected by specific probiotics (Dinan and Cryan, 2013). Intestinal microbiota may change the perception of pain and selective probiotic strains may inhibit the hypersensitivity and intestinal permeability induced by the stress (Gareau et al., 2007; Wiley et al., 2017). Probiotic bacteria manipulate intestinal microbiota, enhancing variety and beneficial composition of the bacteria (Karimi and Pena, 2003). An improvement in microbiota metabolites, such as SCFAs and tryptophan, indirectly improves CNS function (Butler et al., 2019). Studies demonstrated that gut microbiota condition regulates BBB permeability (Braniste et al., 2014). However, the mechanisms underlying these beneficial effects are not well understood yet and, currently, very few human studies are present. Probiotic role may include exclusion of pathogenic microorganisms and immune system modulation (Lavasani et al., 2010; Kwon et al., 2013).

And, most likely, the main proponents of this connecting link are the MCs and their products. The ability of specific strains of bacteria to influence MC function and their activation has been studied, sometimes even with conflicting results (De Zuani et al., 2018).

*Lactobacillus rhamnosus* and *B*ifidobacterium *infantis* probiotic strains reduce depressive-like behavior down- regulating HPA axis (Bravo et al., 2011).

Moreover, it has been reported that *L. rhamnosus* GG and some other probiotic strains show a decreasing effect on the MC numbers in several studies in rodent models (Cassard et al., 2016). Some commensal bacteria can limit MC activation. Pathogenic bacteria, including *Yersinia pestis* and *Salmonella typhimurium*, prevent MCs degranulation in rodents and in humans. This pathogen eludes host innate immunity, involving the MC inactivation (Choi et al., 2013). Both pathogens secrete a tyrosine phosphatase responsible for the suppression of MC activation, leading to a reduced bacterial clearance (Choi et al., 2013; De Zuani et al., 2018). Different commensal bacteria such as *Enterococcus faecalis, Lactobacillus paracasei*, and non-pathogenic *Escherichia coli* can delete MC degranulation IgE/Ag-induced in mice (Choi et al., 2016). It is noteworthy that this suppression mechanism is based on impairment of intracellular signaling including an inhibition of the maintenance of elevated intracellular Ca^2+^ levels required for MC degranulation and not down-regulation of Fc𝜀RI (Kasakura et al., 2014; Cassard et al., 2016). In addition, different strains of Lactobacilli may suppress MC degranulation but not TNF-α release nor IL-13 (Harata et al., 2016). The role of commensal microorganisms in controlling MC activation was evinced following oral treatment of *E. faecalis* that reduced MC infiltration in a murine model (Choi et al., 2016; De Zuani et al., 2018).

The role of bacteria and MCs can also be linked to the beneficial stabilizer agents. It has been shown that the histamine H1 receptor blocker diphenhydramine, known as Benadryl^®^, prevents the increase of cytokines from MCs stimulated by bacteria. Some broad-spectrum antibacterial agents inhibit MC activation and its degranulation. In particular, some probiotic strains are able to stabilize MCs, especially *L. rhamnosus GG* (Oksaharju et al., 2011). Oral administration with *L. rhamnosus JB-1* induces inhibition of peritoneal MC degranulation (Forsythe et al., 2012). The effect is mediated by an inhibition of calcium-dependent potassium current (KCa 3.1). This inhibition prevents Ca^2+^ entry required for MC degranulation. In MC regulation of calcium-dependent potassium current is operated through β2-adrenoceptors and adenosine and prostaglandin receptors (Duffy et al., 2007, 2008). Some Lactobacillus strains are able to inhibit IgE-mediated degranulation in MCs through TLR-2 pathway (Kawahara, 2011). A recent study reports that *L. rhamnosus JB-1* reduced stress-induced behavioral deficits in mice, including modifications in sociability and anxiety. This probiotic prevented immunoregulatory alterations related to the stress phenotype suggesting a direct modulation on gut–brain signaling (Bharwani et al., 2017; McVey Neufeld et al., 2018). Administration of specific bacteria induced systemic expansion of Treg, an immune population that produces anti-inflammatory cytokine, such as IL-10 (Kim et al., 2019).

Another study reports that *L. rhamnosus* GG in combination with prebiotics reduces the effects of early-life maternal separation on anxiety-like behavior and hippocampal-dependent learning with modulation of mRNA expression of genes related to stress circuitry, anxiety, and learning in a rodent model (McVey Neufeld et al., 2017).

A recent study reports that probiotic VSL#3 suppress visceral hyper-sensibility through MC-PAR2-TRPV1 pathway in a rodent model (Li et al., 2018).

Kim et al. (2016) report that treatment with *Bifidobacterium longum* KACC 91563 can control the number of MCs in the gut lamina propria. A modality of intercellular communication is the release of membrane vesicles. Microbiota secrete complex extracellular vesicles (EVs), containing protein, DNA, and components of cellular wall within little spherical lipid bilayer acting as messengers. Bacterial EVs may influence the physiology of neighboring cells, inducing intracellular signaling via receptors and giving new features after the acquisition of receptors, enzymes, or genetic material from the vesicles. Bacterial EVs deliver elements at host immune cells in concentrated, preserved, and targeted manner. In particular, MCs internalize EVs specifically. The authors suggest a model of action according to which EVs of *B. longum* enter through intestinal epithelial cells inducing the apoptosis of MCs (Kim et al., 2016).

*Bifidobacterium longum, B. breve, L. rhamnosus*, and *L. helveticus* have been used and all exhibited antidepressant effects (Desbonnet et al., 2008). *B. longum* and *L. helveticus* suppress stress-related visceral sensibility via HPA axis control (Ait-Belgnaoui et al., 2018). Beneficial effects of a combination of *Bacillus subtilis* and *Enterococcus faecium* (LCBE) could inhibit the degranulation of MCs, confirming the role of probiotics in the regulation of MCs. In addition, the levels of histamine, which constitute an aggravating mediator that accelerates the development of main lesion of organ, were decreased after administration of LCBE (Guo et al., 2019).

It has been shown that an increase in MCs was countered by inclusion of *B. licheniformis* in the diet of a bird animal model. The strain administration partially alleviated stress condition, suggesting that probiotics can benefit intestinal integrity (Deng et al., 2012).

Probiotics influence mucosal MCs, but also could affect MCs in the brain through microbiota–gut–brain axis. *Lactobacillus reuteri* and Bifidobacteria have positive therapeutic effects on cognitive and emotional impairments in fibromyalgia (Roman et al., 2018). Recently, probiotic supplement on inflammatory markers, and episodic and chronic migraine has been reported (Martami et al., 2019).

Short chain fatty acids also have an effect on MC activation (Diakos et al., 2006). Sodium butyrate reduced the percentage of degranulated MCs and their inflammatory mediator content in weaned pigs. In addition, sodium butyrate reduces the expression of MC-specific tryptase, TNF-α, and IL-6 mRNA. A butyrate-producing probiotic (*Clostridium butyricum*) restores the intestinal epithelial barrier integrity through regulation of TWIK-related potassium channel-1 (Trek-1) (Huang et al., 2016).

Also vitamin D is known to play an immunoregulatory role on MCs. In fact, vitamin D is required to maintain stability of MCs (Liu et al., 2017). A deficiency of vitamin D results in MC activation.

In addition, MCs express 25-hydroxyvitamin D-1α-hydroxylase which enables them to convert inactive 25-hydroxyvitamin D3 (25OHD3) to biologically active 1α,25(OH)2D3 (Yip et al., 2014). 1α,25(OH)2D3 favors apoptosis and inhibits maturation of bone marrow-derived MC precursors in mice (Baroni et al., 2007). Vitamin D regulates NO, which is believed to be an anti-bacterial molecule, produced by MCs. Finally, factors that reduce CRF stabilize activation of MCs (Larauche, 2012).

## Conclusion

Mast cells are old cells, perhaps, long forgotten, constitutively and strategically located within the mucosal districts, and armed with a wide variety of receptors that allow them the ability to interact strongly with other cells and elements of the microenvironment. The mutual interactions that regulate the MC–microbiota crosstalk show how MCs act at the crossroad of immune system, intestinal mucosa, commensal microbiota, and nervous system.

The evidence supported the idea that there is likely a bi-directional relationship between MC and intestinal microbiota and that MC activity goes well beyond the simple host defense role of regulating microbial composition during pathology condition.

Most of the data obtained so far are derived from *in vitro* or *in vivo* animal studies because human studies are still very limited.

In this review, we refer some interactions that control MC activation and functions, suggesting that various factors and mechanisms can influence the MC–microbiota–gut–brain crosstalk and regulate their output.

A classical approach detected to prevent improper MC activation has been the use of MC stabilizing agents, including Cromolyn, Ketotifene, and Tranilast, although it is not clear to what extent these drugs act and their exact action mechanism (De Zuani et al., 2018). An increase in intracellular Ca^2+^ concentration as a result of Ca^2+^ entry from the extracellular medium is essential for MC degranulation and Fc𝜀RI-mediated MC activation (Baba et al., 2008) as well as calcium is required for activation of transcription factors of cytokine genes including IL-4, TNF-α, and IL-13 (Heatley et al., 1975; Chu et al., 2016; Zhang et al., 2016). In addition, the validity of MC stabilizers in the treatment of MC-related disorders is as yet uncertain (He, 2004; Wouters et al., 2016).

Dietary ingestion of probiotics and prebiotics affects gut microbiota composition, underlying the key role played by specific metabolites not only in the gut microbiota composition but also in the brain health state. Some probiotic effects and mechanisms can be similar to those achieved by drugs and may provide suggestion to future interventions.

Indicating strategies preventing MC activation are very more interesting because, once activate, MCs lead perpetuate inflammation state through onset positive feedback loops.

## Author Contributions

GT researched and summarized the information and wrote the review.

## Conflict of Interest Statement

The author declares that the research was conducted in the absence of any commercial or financial relationships that could be construed as a potential conflict of interest.
